# Intercity Mobility Assessment Facing the Demographic Challenge: A Survey-Based Research

**DOI:** 10.3390/ijerph20021163

**Published:** 2023-01-09

**Authors:** Juan Miguel Vega Naranjo, Montaña Jiménez-Espada, Francisco Manuel Martínez García, Rafael González-Escobar, Juan Pedro Cortés-Pérez

**Affiliations:** 1 Department of Construction, Research Institute for Sustainable Territorial Development (INTERRA), School of Technology, Universidad de Extremadura, Avda de la Universidad s/n, 10003 Cáceres, Spain; 2 Department of Art and Territorial Sciences, Universidad de Extremadura, 10003 Cáceres, Spain

**Keywords:** rural areas, demographic challenge and depopulation, motive for displacement, patterns of displacement mobility, accessibility to basic services

## Abstract

The key factor in moving towards a more sustainable travel model is based on improving mobility, especially in rural areas that share territorial dynamics with urban areas and are connected by a daily flow of inhabitants. The purpose of this article is to carry out a diagnosis of the daily mobility patterns of the inhabitants of a number of rural municipalities, with the aim of promoting sustainability and mitigating the phenomenon of territorial depopulation in future local planning policies. The research methodology is based on the use of revealed preference surveys together with accessibility analysis using GIS tools, allowing for an in-depth knowledge of the mobility patterns of the municipalities in the area under analysis. In this respect, the reference parameters in terms of territorial accessibility are determined by applying the network analysis procedure to basic public services. The results reflect the existence of an unbalanced modal split with a preponderance of private vehicle use (regardless of the destination or the reason for the journey). In addition, a very weak inter-municipal connection dynamic is observed. There is a knowledge gap in the verification of the long-term suitability of sustainable measures in rural areas implemented after the development of mobility plans (in order to assess their effectiveness).

## 1. Introduction

The 21st century is now recognised as the century of cities. The population dynamics that originated in the 19th and 20th centuries continue today to favour the settlement of the population around well-defined urban poles. At the same time, some rural or small urban areas are in the process of depopulation or demographic stagnation [1]. However, changes in land use, new infrastructures, purchasing power, the ageing of the population or the perception of the inhabitants maintain the need to implement social, economic and environmental sustainability policies at all territorial scales, as well as to improve and extend existing ones.

One of the most important areas when it comes to improving the sustainability of our territories is mobility, with the fact that pollution caused by urban transport continues to rise compared to other sectors that manage to mitigate their emissions [2]. In other words, there is a need to address the mobility of those areas (whether rural, urban or metropolitan) that share territorial dynamics or, in general, that are connected by a daily flow of inhabitants between their different built spaces [3,4]. Therefore, in addition to aiming to limit mobility needs by generating compact and dense urban cores, it is also essential to achieve a shift towards sustainability in existing mobility flows. In this sense, many cities have been implementing measures for years to promote a modal shift from motorised vehicles to sustainable and active forms of transport [5,6,7,8,9], with the aim of achieving the positive ecological, health and economic impact reported by several studies [10,11,12]. In any case, the creation of sustainable mobility infrastructures seems to have a disparate impact depending on different factors beyond distance and time, such as their urban or interurban character [10,13,14,15], the quality or aesthetics of the environment [16,17], perceived safety [18,19], the dynamism associated with the spaces [20], and the familiarity of users with the proposed alternative modes [21,22].

Thus, the population dispersion of Spanish urban environments has led to an increase in mobility in private vehicles and an increase in travel distances [23]. Different projects have been carried out with the aim of alleviating the problems associated with congestion in urban centres, which is a phenomenon that has led to environmental deterioration associated with traffic congestion problems in those areas that are a factor of socioeconomic development [24,25] and which are associated with continuous population movements in which the use of private vehicles predominates.

With the aim of improving mobility in urban environments, a series of technical studies have been carried out, both municipal and supra-municipal, called Sustainable Urban Mobility Plans (SUMP), which include a set of actions that aim to implement more sustainable forms of travel in urban areas with the aim of reducing energy consumption and pollutant emissions, thus guaranteeing the quality of life of citizens, achieving social cohesion and economic development [26].

Different studies on urban mobility show that there are success stories on the road to sustainable mobility, although in Spain, they are limited to specific cities such as Vitoria-Gasteiz, Seville, Barcelona, Valencia, Valladolid and Pontevedra [6,27]; these successes are attributable not only to the political action taken by their city councils but also to the social consensus generated. These achievements are a source of hope for cities intending to implement sustainable mobility measures, as their experiences can shed light on many issues and provide an example of which measures could be more effective a priori.

However, these cities have been limited in their ability to move such changes outside their urban core and the surrounding rural, urban and metropolitan areas continue to be dominated by private motorised transport [13,28]. In this sense, there is a perceived need to start implementing sustainable mobility plans coordinated with supra-municipal authorities, so that sustainable mobility policies can reach beyond the boundaries of central municipalities. One of the current challenges is to analyse daily mobility in functional areas (consisting of a city and its surrounding municipalities), so that it can be addressed with quantified and measurable data, enabling a monitoring of the mobility of the population and being able to assess which measures to implement, their feasibility, impact and efficiency. Some studies have analysed the potential for modal shift in metropolitan areas towards public transport, traditional and electric bicycles [29,30,31], quantifying the origins and destinations of mobility, the reasons for choosing their current mode of transport [11,32] and the impact of possible solutions in low-density rural environments [33,34,35]. Other surveys and interviews have been conducted using qualitative and quantitative methodologies to analyse users’ perceptions of infrastructure quality and use [36,37]. In general, these studies indicate that the existence of quality transport services and networks as an alternative to the private car has great potential to attract new users, although there are certain sectors of the population that seem to resist alternative modes to the car for social reasons, such as prestige or identification with a high economic position [38,39,40]. In contrast, studies in Germany have found different results, linking higher social status with higher bicycle use [41]. In addition to the differences observed on the basis of social class, other surveys have found disparities in modal split by gender and age [42,43,44], many of which can be solved by making infrastructure attractive to all users [45].

On the other hand, ref. [46] seeks to quantify the effects of walkability on reduced mobility and active travel associated with the ageing process. Living in poorly walkable areas places older adults in a critical situation that often leads to less healthy travel patterns. In [47], it is demonstrated how exposure to heavy traffic negatively affects children’s perceptions of their environment and how improving the pedestrian environment (constructing walkways and improving a pedestrian crossing) can change those perceptions.

From an environmental point of view, the findings of ref. [48] confirm existing evidence on the health benefits of urban greenery and extend the analytical focus on the impact of green space provision on the physical health and well-being of urban residents. The presence of green spaces is found to increase walking levels, which is of significant relevance in the context of ageing modern societies [49].

In relation to more sustainable travel patterns, ref. [50] addresses an integrated approach to transport and urban planning, which aims to reduce car use and urban sprawl, increase the use of public transport and improve sustainable mobility. The strategy would enable its inhabitants to have a healthy lifestyle (improved physical activity) and reduce traffic congestion (greenhouse gases) as well as mental health disorders (driving stress). In parallel, ref. [51] conducted a systematic literature review identifying essential indicators to quantify the sustainability of transport systems, with a particular focus on urban mobility. This study could be useful in assisting researchers exploring the evaluation of sustainability perspectives in urban transport systems.

Finally, the authors [52,53] examine how urban transport influences the achievement of social sustainability in urban regions, which is implicit in the literature through concepts such as social equity, social exclusion and quality of life [54]. The lines of research analyse aspects such as urban form, location- and individual-based accessibility, and urban/regional planning for sustainability, uncovering current paradigms, key research areas and the link between the fields of study of sustainable transport infrastructures. In order to achieve sustainable urban development through strategies that establish integrated measures to address the economic, environmental, climatic, demographic and social challenges affecting urban areas, while taking into account the need to promote links between urban and rural areas, the Provincial Council of Cáceres launched the drafting of a Sustainable and Integrated Urban Development Strategy (EDUSI) composed of the city of Cáceres and its 21 neighbouring municipalities forming an area with strong links [55]. As a result of the implementation of this strategy, the Sustainable Urban Mobility Plan (SUMP) for the interurban area of the municipality of Cáceres is being drafted with the aim of implementing the challenges identified by society through a process of citizen participation in the field of mobility and transport. These plans should serve as a basis for establishing a sustainable model of local mobility between the municipalities that make up the area, thus enabling economic development, environmental conservation and social welfare [56,57,58].

The aim of this article is to quantify and analyse the usual mobility of rural inhabitants of the Network of Sustainable Municipalities of Cáceres, which is an urban area established by the Provincial Council of Cáceres and made up of 21 municipalities whose mobility dynamics are mainly related to the provincial capital. While the city has urban mobility data collected in 2014, the rural and interurban mobility of the functional area has not been analysed until now. The evaluation of data by gender, age and municipality, related to other sociological variables such as studies, employment situation, home, and family, as well as the availability of means of transport and the daily habits of the population, allows us to carry out a diagnosis of the interurban mobility patterns in the area under study.

The importance of this research lies in the researchers’ concern with improving the quality of daily life of the inhabitants living in the RMSC. To this end, a global analysis of mobility patterns and the degree of territorial accessibility to health and education centres (among others) is developed, using GIS tools in combination with revealed preference surveys. The main purpose of the study is based on a diagnosis of the daily interurban travel habits of the inhabitants of the RMSC, with the hypothesis of serving as a basis for the establishment of a sustainable model of local mobility between the municipalities that make up the study area, thus enabling economic development, environmental conservation and social welfare. There is a knowledge gap in mobility studies in rural areas with a practical application of sustainable solutions that present a follow-up and traceability of the measures in order to evaluate their effectiveness.

## 2. Materials and Methods

The methodology developed in the research consists of three distinct parts: (1) conducting a revealed mobility survey, (2) obtaining baseline data by searching institutional repositories with open access information, and (3) applying geographic information system tools. The revealed mobility survey, referred to as the revealed preferences (RP) method, is one in which information about the actual behaviour of individuals is collected [59,60], and in this way, problematic aspects related to mobility in the municipalities of the study area can be located. The field data collection work for the development of the survey was carried out between May and July 2021, and the values obtained were processed during the following 6 months. It can be seen that during this period of time, no infrastructure relevant to mobility has been built in the study area, nor has any campaign been carried out to promote modal shift. The data collected are therefore valuable information for public authorities, as they are an essential starting point for spatial planning. The quantitative methodology based on surveys is supported by its widespread use in mobility studies, and its use additionally allows the chronological monitoring of the studied variables [36,61,62,63].

**Figure 1:**
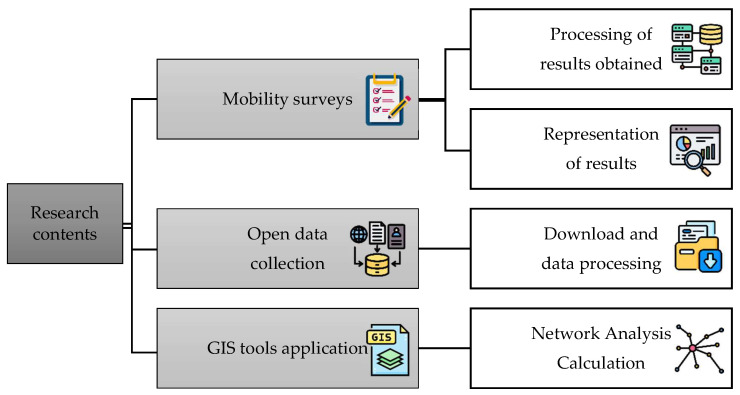
Methodology flowchart.

To complete the study, in addition to the sample used, we have also used population data by sex and age grouped into five-year groups from the Statistics section of the continuous census as of 1 January 2021, which have been obtained from the National Statistics Institute (INE), as well as data on the active population by sector. With reference to the cartographic layers, they have been extracted both from the Download Centre of the National Geographic Institute (CNIG) and from the download centre of the Territorial Information System of Extremadura (SITEX). With the data obtained from the different institutional repositories, analyses have been carried out to gain a deeper understanding of the situation of the study area in terms of sociodemographics, economics and mobility. Finally, in order to develop the accessibility analysis of the study area, Geographic Information Systems (GIS) techniques were used using the network analysis tool [64,65,66] incorporated in ArcGis’ ArcMap software. Figure 1 shows the flow diagram of the methodology used in the research:

### 2.1. Study Area

**Figure 2:**
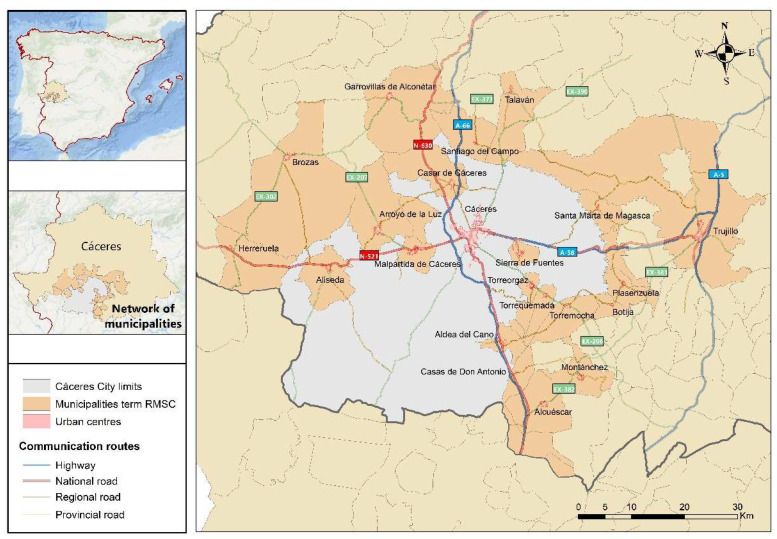
Location map of the study area.

In order to address the daily mobility of the rural inhabitants of the Network of Sustainable Municipalities of Cáceres (RMSC), the study area defined in Figure 2 is established. It can be seen that the RMSC is composed of those municipalities of the province that border the municipality of Cáceres. This city and its surroundings have in common the pendular movements that are practised from the surrounding municipalities towards the provincial capital (the true socioeconomic, administrative and commercial axis of this territory).

This study area is partially made up of some of the municipalities that are defined within the functional urban area based on the INE definition of the city of Cáceres (Aliseda, Arroyo de la Luz, Casar de Cáceres, Garrovillas de Alconétar, Malpartida de Cáceres, Sierra de Fuentes and Torreorgaz), in addition to another series of adjacent municipalities, which justifies the existing interrelationships between municipalities.

The city of Cáceres [67] is the main travel attractor, which is located in the centre of Extremadura and geographically bordering the 21 municipalities that make up the RMSC. The area under study is structured by an outstanding network of communication routes that facilitate mobility between municipalities. These include the A-66, which runs the length of the province (from north to south) and the A-58, which connects the city of Cáceres with the A-5. In addition, it is necessary to mention the structure of the network of national and regional roads which link the most dynamic municipalities in the RMSC with the city of Cáceres (a real hub for the development of the surrounding municipalities). Finally, it is worth mentioning the road network of the Cáceres Provincial Council, which guarantees the branching of connections with higher capacity networks and allows interconnections between municipalities with smaller populations.

In terms of railway infrastructures, the offer is almost exclusively limited to the service provided by the Madrid-Cáceres-Badajoz line which connects the study area with Mérida and Badajoz to the south, and with Plasencia, Talavera de la Reina and Madrid to the northeast, but which is of little use for interurban communication in the study area due to the lack of routes and stops.

**Figure 3:**
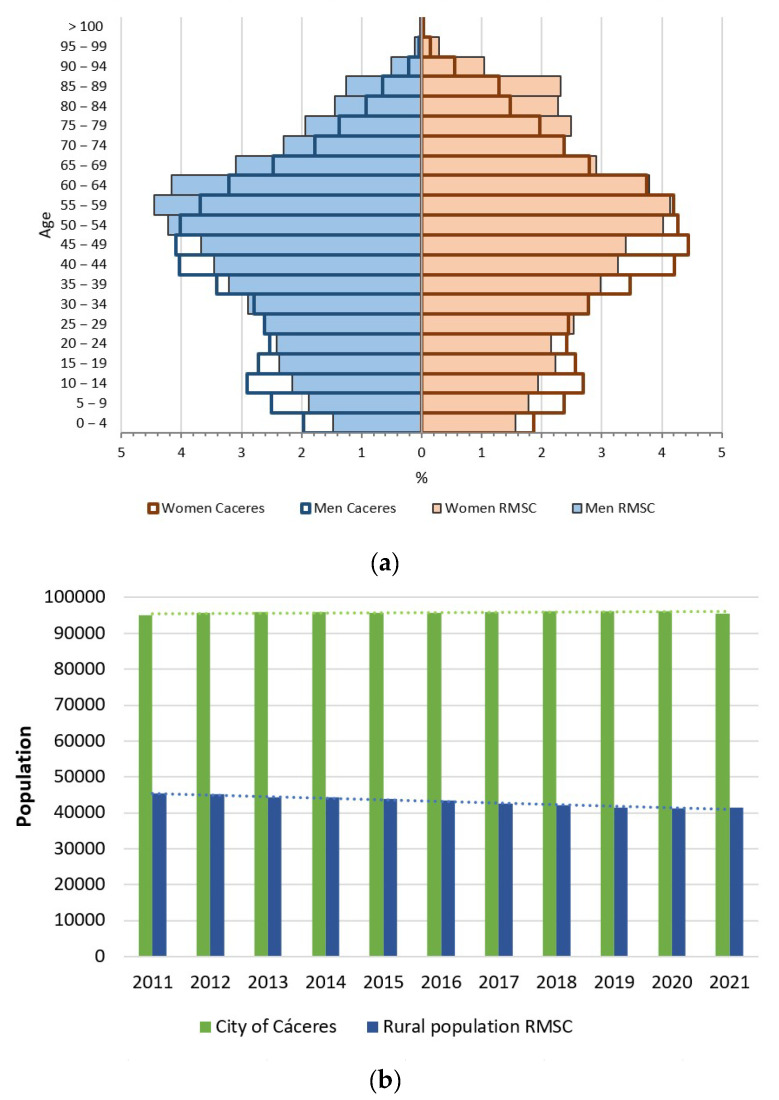
Comparison of the population situation of the study area and the city of Cáceres: (**a**) Population pyramid of Cáceres and the RMSC; (**b**) Evolution of the population of Cáceres and the RMSC 2011–2021.

As for the description of the sociodemographic characteristics, Figure 3a shows a comparison of the population situation both in the city of Cáceres and in the surrounding rural areas; in turn, Figure 3b shows the evolution of the demographic data for the last 10 years.

Figure 3a shows the clear existence of a narrowing at the base of the population pyramid as well as a widening in the age groups from the age of sixty onwards. This fact determines how in specific territories, a continuous ageing of the population is taking place, and it is a latent problem that is increased in rural populations (where this effect is more noticeable in its structure). The city of Cáceres, which has a population of around 95,000 inhabitants, has undergone slight variations in the last decade, but it has managed to maintain a population of close to 100,000 inhabitants. However, the rural municipalities of the RMSC have suffered a significant drop in population in recent years, although they have shown slight increases in population associated with periods of crisis, as is the case of COVID-19.

It should be noted that the study area has a population of 136,798 inhabitants (data obtained on 1 January 2021), representing 35.12% of the total population of the province of Cáceres. Of the figure previously provided, 41,380 belong to the 21 rural municipalities bordering Cáceres (equivalent to 30.25% of the total population of the study area excluding the capital), in which three groups of municipalities have been established according to population size (Table 1). Despite accounting for one-third of the population in relation to the total population of the area analysed, this population is the most challenging in terms of providing sustainable mobility solutions, both due to its location and its degree of ageing.

With regard to strictly economic aspects, it can be seen that the population of the RMSC works in the tertiary sector (89% in the city of Cáceres and 63% in the neighbouring municipalities), which is the sector with the greatest weight in the economy of the territory. Similarly, it is clear that the primary sector (8% of workers) and the secondary sector (29% of workers) [68] are of little importance in the rural populations of the study area. In contrast, there is the city of Cáceres, whose active population is barely present in the aforementioned sectors, with values close to 2% in the case of the primary sector and slightly above 8% in the secondary sector.

### 2.2. Methodology for Calculating Accessibility in the RMSC (Network Analysis)

With the premise of calculating the different accessibility parameters in the study area (RMSC), we start from the research developed in [69,70,71] where network analysis is established as the main study tool in order to determine citizen mobility and accessibility to the main basic services. In this sense, we proceed to elaborate a matrix containing information on the duration of trips from the origin (21 rural municipalities in the study area) to the destination (city of Cáceres, as well as to health centres and secondary education centres located in the study area) as established in [64,72], which uses the network analysis tool for studies of accessibility to health centres in Extremadura (Spain) and to companies located in India. The selection of destinations considered in the analysis is based on two aspects: (1) access to the city of Cáceres is essential in order to determine the mobility patterns of the municipalities of the RMSC and (2) taking as a reference the legislation of the autonomous community of Extremadura on land use planning [73,74], it is very useful to determine the accessibility of public facilities and basic services to reach each population centre in the study area. Thus, based on the previously described matrix, we have chosen to generate an isochrone map that allows us to evaluate the travel time from the rural municipalities of the RMSC to the city of Cáceres, using the private vehicle as a means of transport via the main roads in the study area (roads, motorways and junctions).

For the study of accessibility to the main services in the study area, the graph theory has been used, this being a collection of nodes, which correspond to the centroids of each population centre and the established points of interest in the city of Cáceres (health centres and secondary education centres), which are connected by edges (all the communication routes). Subsequently, the minimum travel time from each population centre to the nearest point is calculated, knowing the hierarchy of the network and based on the value of the impedance (a fundamental factor in the study of accessibility). For this purpose, the impedance has been obtained from the length (in km) and speed (km/h) of the roads (considering the maximum speed allowed on each type of road) by means of the following expression:

[Length/Speed] × 0

The value of the impedance parameter has been established in minutes, considering the time taken by a private vehicle (in this case a car) to travel each section of the road network.

With the above information, the closest route is calculated by means of network analysis using the tools of the ArcGis ArcMap software, obtaining the route and the minimum time taken to travel from each population centre to the facilities described above.

Then, in order to determine the minimum travel time, the IDW (weighted inverse distance) was used, which is a method that allows the interpolation of cell values by means of the weighted combination of a set of points to determine the inverse distance of these values. The inverse weighted distance equation or “Shepard method” is as follows:

$$F\left(x,y\right)=\overset{n}{\underset{i-1}{\sum }}w_{i}f_{i}\;$$

where *n* is the number of scatter points in the set, *f_i_* are the values of the functions set at the scatter points and *w_i_* are the weighting functions assigned to each scatter point. The weight formula:

$$w_{i}=\frac{h_{i}^{-p}}{\sum_{j-1}^{n}h_{j}^{-p}}$$

where *p* is an arbitrary positive real number called the power parameter (typically, ^*p*^ = 2) and hi is the distance from the scattering point to the interpolation point, or:

$$h_{i}=\sqrt{{\left(x-x_{i}\right)}^{2}+{\left(y-y_{i}\right)}^{2}}$$

where *x* and *y* are the coordinates of the interpolation point and *x_i_* and *y*_i_ are the coordinates of each scattering point [64].

The surface that is interpolated has to be a location-dependent variable. Thus, the use of this method tolerates that the variable that is represented cartographically has a decrease in its influence related to the location of the sample the further away it is from the sample. Therefore, it can be seen that the best results obtained from the IDW method are when the sample is dense with respect to the local variation to be simulated, so that those point samples that are dispersed will not represent the desired surface in the results. Therefore, to determine the accessibility of the population centres of the study area, this method is quite effective, using the variable that determines the minutes from one point to another, with the intention of knowing the minimum travel times from the centres to the city of Cáceres, to the health centres and to the secondary schools of the RMSC.

### 2.3. Length, Procedure and Survey Structure

The period for carrying out the revealed preferences survey ran from May to July 2021. During this period, surveys were first carried out in person (on paper) in the municipalities belonging to the SUMP. At the same time, web links to the survey were sent to the different competent public administrations and neighbourhood associations belonging to these municipalities for the development of online surveys. Likewise, a QR code was enabled so that the inhabitants of the study area could conveniently carry out the surveys on their mobile devices.

In relation to the content and structure of the survey, a first block was established that alluded to the personal data of the respondents. This block was intended to facilitate the segregation of respondents according to various considerations (gender, age, municipality of residence) with the intention of being able to filter the data and determine mobility between municipalities in the study area. The second block provides information on the respondents ’ household data, referring to the access of the children in the household to educational centres, if any, or the type and number of vehicles in the household.

The third block of questions deals with understanding mobility habits in the study area. At the beginning of this block, questions are asked about the means of transport used and the destination and frequency according to different categories, typologies or motivations (work, studies, shopping, health reasons, leisure and administrative formalities). Subsequently, questions are asked about timetables, duration of journeys, vehicle parking or number of occupants. In this way, the purpose of this block is to gather information on the type of mobility in the study area and the different problems that may exist during these journeys.

### 2.4. Sample Size

In order to carry out the opinion poll, a total of 294 valid surveys were carried out for a total of 36,909 inhabitants (population over 18 years of age) in the study area, which means a margin of error of approximately ± 5%. Table 2 shows the typology of the people surveyed classified according to: gender, age, employment status, duration of trips and use of public transport.

In order to calculate the margin of error of the surveys carried out and to validate them, the following formula was used:

(1) $$n=\frac{N\cdot z2\alpha 2\cdot p\cdot \left(1-p\right)}{e2\cdot \left(N-1\right)+z2\alpha 2\cdot p\cdot \left(1-p\right)}$$

The values used for the calculation of the margin of error are as follows: t-size of the RMSC population N = 36,909; sample size n = 294, with a distribution of p = 0.5. Therefore, the confidence level is 95% (1−α), so the value zα/2 = 1.96. As a result of this calculation, it is concluded that the margin of error is within ± 5%.

## 3. Results

The results obtained in the research are offered in two complementary ways: (1) on the one hand, an accessibility study based on network analysis is developed with the premise of gaining in-depth knowledge of interurban mobility in the rural municipalities that make up the RMSC, (2) after this first analysis, the information collected in the revealed preferences surveys is used, where the responses obtained by citizens on mobility derived from the public participation process are analysed.

### 3.1. Analysis of the Accessibility of the RMSC Using Geographic Information Systems (GIS)

#### 3.1.1. General Accessibility of the Rural Municipalities of the RMSC to the City of Cáceres

**Figure 4:**
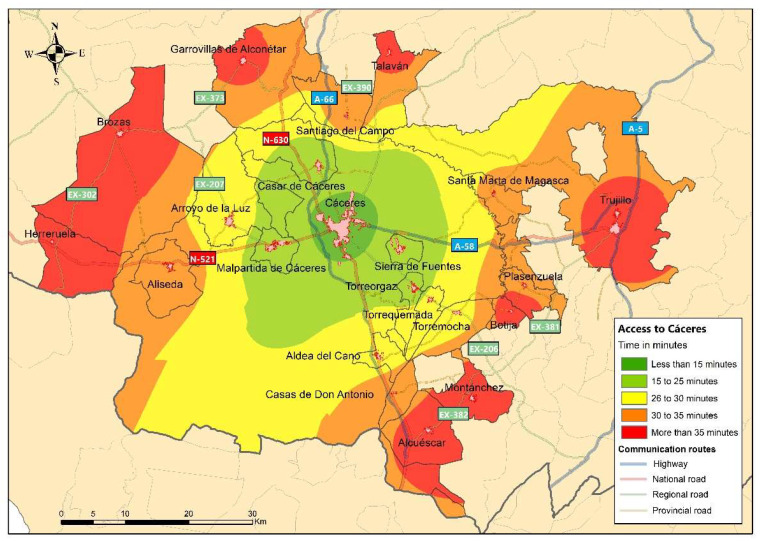
Territorial accessibility to the city of Cáceres.

In order to evaluate the flow of inhabitants from rural towns to the provincial capital, an analysis of territorial accessibility is carried out, initially considering the use of private vehicles as a means of transport, as can be seen in Figure 4. A perimeter ring is formed around the urban centre of Cáceres, where access time is less than 15 min. The next range considered, with an access time of between 15 and 25 min, is made up of the municipalities closest to the city of Cáceres, which are connected to national roads in the case of Malpartida de Cáceres and Sierra de Fuentes (N-521), regional roads such as in the case of Torreorgaz (EX-206) or provincial roads such as those which connect to Casar de Cáceres (CC-38). Still within the area of influence of the city of Cáceres, we find municipalities such as Arroyo de la Luz, Torrequemada, Torremocha and Aldea del Cano, which show slightly longer access times in relation to their geographical location (between 25 and 30 min). The municipalities of Aliseda, Santiago del Campo, Santa Marta de Magasca, Plasenzuela and Casas de Don Antonio have an access time to Cáceres of between 30 and 35 min, which is mainly due to the fact that they are located at a greater distance and due to the lack of more direct communication routes between origin and destination. The rest of the municipalities (Herreruela, Brozas, Garrovillas de Alconétar, Talaván, Alcuéscar, Montánchez, Botija and Trujillo) have a longer access time to Cáceres (more than 35 min), which is due to the fact that they are the municipalities furthest from the destination, even though they are located next to high-performance communication routes (motorways), as is the case of Alcuéscar and Trujillo.

As we will analyse later, it can be seen that the city of Cáceres acts as a real pole of population attraction for various reasons of travel, causing the rural inhabitants of the municipalities in the study area to make daily trips with a duration that, in many cases, could exceed 30 min. However, it can be observed that the duration of these journeys is considerably reduced by the possibility of using high-performance communication routes, as is the case of those municipalities which are closer to the A-66 and the A-58. For this reason, accesses to the city of Cáceres from the different points in the study area have a relatively short journey time, as the study area is linked by important national and regional roads.

#### 3.1.2. Accessibility of the RMSC to Health Centres

In terms of territorial accessibility, the second significant aspect under analysis in the interurban mobility of the RMSC is the access time to the nearest health centres, considering that the displacement is carried out through the use of a private vehicle. When defining accessibility to health centres, it should be taken into account that there are municipalities in the study area that have a health centre in their own locality and other towns that lack this health service and have to travel to another municipality in the network. There are also some municipalities in the RMSC that depend on health centres located outside the study area, such as Brozas and Garrovillas de Alconétar (which depend on the Navas del Madroño health centre), Herreruela (which depends on Salorino) and Torremocha (which depends on Valdefuentes).

**Figure 5:**
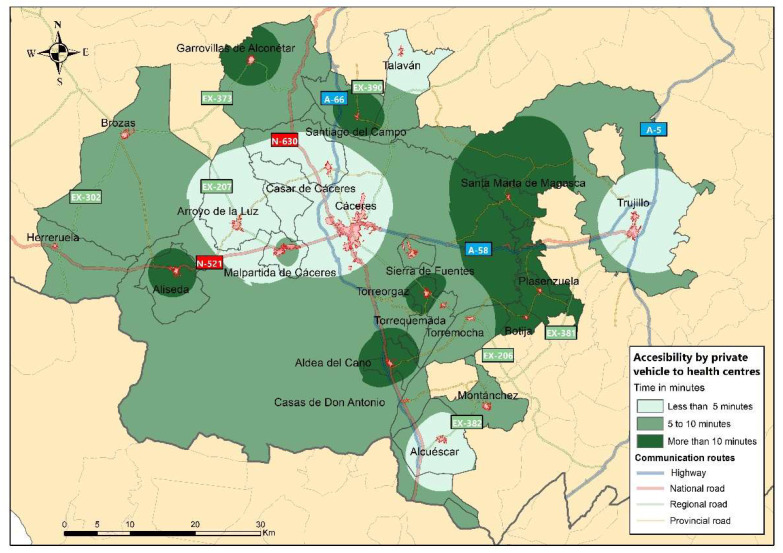
Territorial accessibility to health centres.

As can be seen in Figure 5, three ranges are established according to the access time to the health centres: (1) on the one hand, those municipalities that are less than 5 min away, (2) municipalities with access times of between 5–10 min and (3) those with access times of more than 10 min.

Arroyo de la Luz, Casar de Cáceres, Talaván, Alcuéscar and Trujillo, municipalities that have health centres in their own town centres, are in the first range shown in Figure 5. Of the aforementioned localities, all of them have a population of over two thousand inhabitants, with the exception of Talaván. In the second category, it is necessary to highlight the cases of Malpartida de Cáceres and Sierra de Fuentes. These municipalities have more active socioeconomic and demographic dynamics compared to other municipalities in the study area, and their access time to health centres is slightly longer compared to municipalities with the same characteristics [65]. This is due to the fact that they do not have their own health centre and have to travel to health centres in the city of Cáceres. The third range is made up of municipalities that do not have their own health centre: a first group randomly distributed throughout the study area (Garrovillas de Alconétar, Aliseda, Aldea del Cano, Torreorgaz and Santiago del Campo) and a band located to the east made up of the municipalities of Santa Marta de Magasca, Plasenzuela and Botija.

It is necessary to emphasise that having good territorial accessibility to the health centres of the RMSC is a crucial aspect for the well-being of the citizens, considering the marked ageing of the population resident in the study area. It can be seen that access time is reduced in most cases, slightly exceeding 10 min in the highest values. However, it can be seen that in this third range are located those municipalities with smaller populations and whose demographic characteristics mean that their inhabitants have a greater need for access to these services.

#### 3.1.3. Accessibility of the RMSC to Secondary Schools

The third important aspect considered in the analysis of interurban mobility in the RMSC is the time taken to reach the nearest secondary schools, considering that the journey is made by car. As in the previous case, there are educational facilities within each municipality, either in a nearby municipality within the RMSC or, on the contrary, in a locality that does not belong to the study area but which is attended by students from the network (as in the cases of Brozas and Herreruela). Accessibility to secondary schools is considered a key element for the sociodemographic development of the rural municipalities in the study area, as it facilitates the revitalisation of these areas and their subsequent development [67,68].

**Figure 6:**
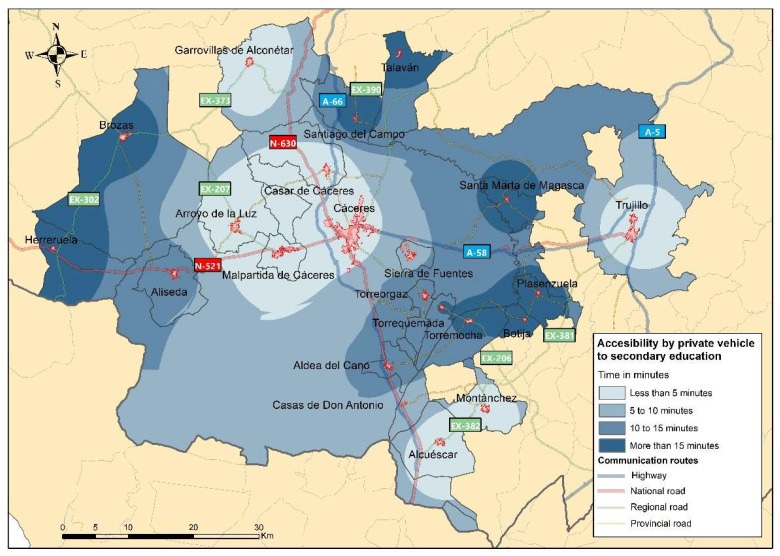
Territorial accessibility to secondary schools.

Figure 6 shows that the municipalities in the RMSC that have their own schools (Alcuéscar, Arroyo de la Luz, Cáceres, Casar de Cáceres, Garro-villas de Alconétar, Malpartida de Cáceres, Montánchez and Trujillo) have travel times of less than 5 min. In the intermediate ranges (5 to 10 min and 10 to 15 min), there are only four municipalities (Sierra de Fuentes, Aliseda, Aldea del Cano and Torreorgaz). On the other hand, there are three distinct locations in the study area where the municipalities have access times of more than 15 min: (1) to the west are Herreruela and Brozas; (2) to the north (Santiago del Campo and Talaván) and (3) to the east between Cáceres and Trujillo (Torrequemada, Torremocha, Botija, Plasenzuela and Santa Marta de Magasca).

It is logical that the location of secondary education centres in the study area is mainly in the municipalities with the largest populations, and these have the shortest access times. On the other hand, the municipalities with a smaller number of inhabitants and with more precarious communication routes show travel times of more than 15 min, with values that can be considered not excessively high for these journeys, which have a daily frequency of more than 15 min.

### 3.2. Preference Poll Results Revealed

The city of Cáceres acts as a real development pole both for the study area and for practically the whole of the province due to its capital, bringing together an important number of administrative facilities at different levels. This fact justifies the significant interurban mobility to Cáceres, the real functional nucleus that extends throughout the area comprising the RMSC. Although at the administrative level, the importance of Cáceres on the territory is very notable, it is also necessary to highlight the significant role that this municipality plays at the educational, employment, health and commercial levels. The results obtained from the surveys carried out show that there is a significant number of trips for reasons related to administrative, health or educational procedures between the towns of the RMSC. At the same time, users sometimes take advantage of this journey to carry out other actions, mainly commercial and, to a lesser extent, leisure activities. In this research, journeys have been classified according to their main motivation, bearing in mind that their final use can be multiple.

#### 3.2.1. Means of Transport Used

**Figure 7:**
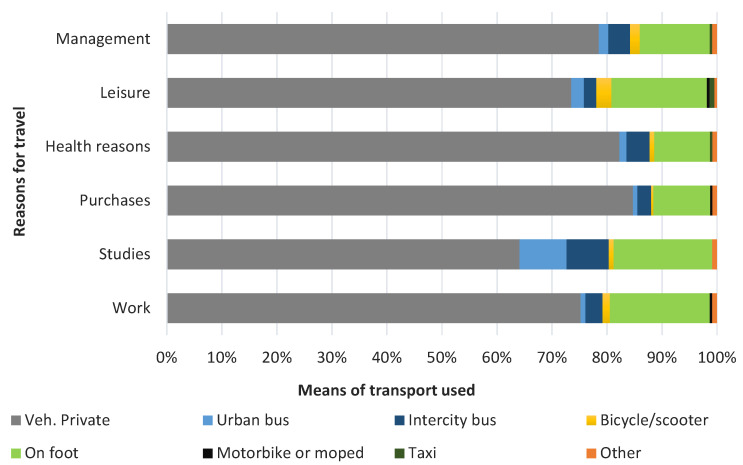
Means of transport used according to travel reasons.

Identifying the means of transport used when travelling within the RMSC is an important issue in terms of interurban mobility, since it refers to how this mobility is developed, what impact it could generate on the environment, as well as what would be (in an estimated way) the occupation of the road. The predominance of the use of private vehicles in the daily life of citizens is palpable in today’s society and way of life, and in the case of the study area, it is very relevant among those surveyed. Figure 7 shows that this means of transport accounts for 76% of journeys made within the framework of the RMSC, which is a very high percentage considering the demographic characteristics of these municipalities and their urban morphology. On the other hand, walking is one of the soft means of transport which is to be prioritised in the RMSC, becoming the second most used means of transport (although it only represents 15% of the total number of journeys). The rest of the means of transport analysed in the study area represent only a token representation.

The use of different means of transport according to the different reasons for travel is again evident in the massive use of private vehicles in each of the categories surveyed, with the highest values being found in the case of shopping and health reasons (84% and 82% respectively), and the lowest when the reason is for study (63%).

Among the data collected, it is worth noting that the main motivation for using the intercity bus is for study reasons, as a large number of students use this means of transport to travel, although the percentage is only 15% of the total.

#### 3.2.2. Frequency of Travel

**Figure 8:**
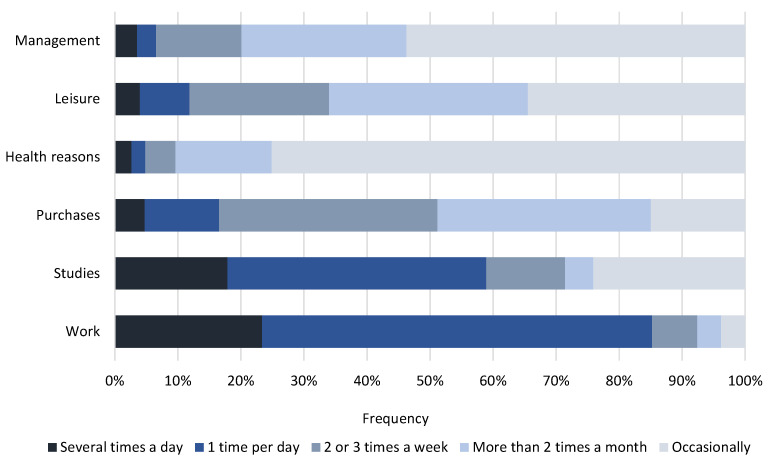
Frequency of trips by purpose of travel.

Another of the aspects evaluated was the frequency of trips according to the activities that motivated them. Figure 8 shows the trips made and allows us to categorise them into two established typologies: (1) trips made for obligatory mobility (work or studies) and (2) occasional or discretional trips (shopping, health reasons, leisure and business). It can be seen that mobility for work reasons accounts for 68% of the total number of people surveyed, for study reasons (36%), shopping (82%), health reasons (74%) and leisure (65%). On the other hand, work-related trips are made almost daily (62% of those surveyed said that they travel once a day for this reason and 23% said that they travel several times a day). For a frequency of several times a week or less, a percentage of 15% corresponds approximately to the percentage of employees who, according to the results of the survey, would telework.

For journeys made for study purposes, there is again a higher proportion of those who make a journey once a day (41% of respondents). It can also be seen that 24% of citizens make journeys for study purposes occasionally and those surveyed who make such journeys several times a day represent 18%.

With regard to the rest of the established categories, there is no clear trend or daily periodicity. In any case, trips for shopping or leisure purposes tend to be made on a weekly or almost weekly basis (around 70% and 54%, respectively).

#### 3.2.3. Connectivity of Municipalities by Population Range

Due to the varied number of municipalities present in the study area and their palpable differences in terms of socioeconomic and demographic characteristics, it is considered necessary to develop the analysis of mobility, taking into account the size of the population. This makes it possible to group the municipalities by population size, analysing the predominant differences and determining the existence of dependency relationships between the smaller and larger municipalities, either among themselves or with the city of Cáceres (the real focus of travel in the study area).

**Figure 9:**
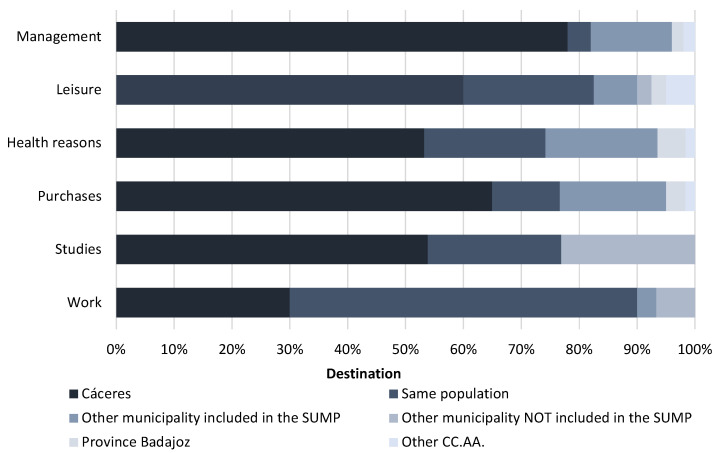
Destination of journeys of municipalities with a population of less than 500 inhabitants according to reason.

In this sense, firstly, we have chosen to analyse the data obtained in the surveys on the destination and the reasons for travel in those municipalities with a population of less than 500 inhabitants (Figure 9), namely: Botija, Casas de Don Antonio, Herreruela, Plasenzuela, Santa Marta de Magasca and Santiago del Campo. According to the results obtained, in the municipalities in this population range, dependence on the city of Cáceres is around 59%, which is followed by commuting within the same population with 20% and commuting to other municipalities in the RMSC with 13%.

Looking more deeply into the reasons for these journeys, it can be seen that trips to the city of Cáceres show values of over 50% in aspects related to study, shopping, health, leisure and business. It is worth noting that 78% of the trips for business purposes are to the provincial capital, which is mainly due to the existence of different state and regional public administrations in Cáceres, as well as to a greater offer in terms of private business (consultancies, medical consultations, etc.) in comparison with the municipalities of origin, being non-existent in municipalities with less than 500 inhabitants. On the other hand, 60% of the reasons for commuting due to work are identified in municipalities with less than 500 inhabitants, which may be due to the economic characteristics of these municipalities, where the main economic activity is related to the primary sector, being agro-livestock farming towns (due to this fact, most of the population has a job in the same municipality).

**Figure 10:**
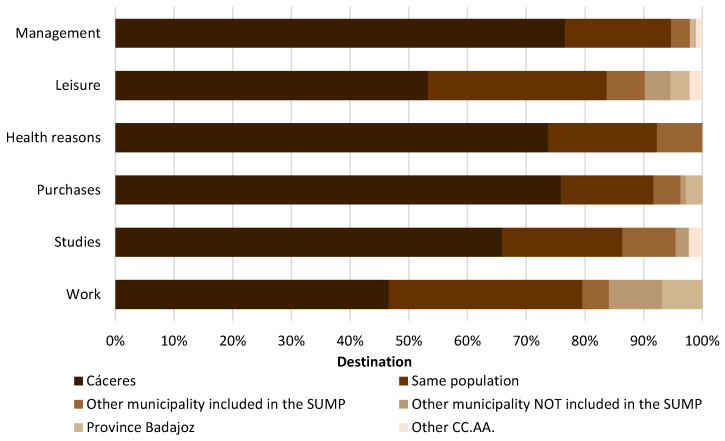
Destination of journeys of municipalities with a population between 500 and 2000 inhabitants according to reason.

Secondly, a new group of municipalities with a population between 500 and 2000 inhabitants has been established (Figure 10) comprising: Aldea del Cano, Aliseda, Bro-zas, Montánchez, Sierra de Fuentes, Talaván, Torremocha, Torreorgaz and Torrequema-da. In terms of the main destination of journeys, the city of Cáceres shows a percentage of 66%, which is higher than that shown above in the group of municipalities with a population of less than 500 inhabitants. This justifies the fact that there is a greater dependence on the provincial capital in the case of municipalities with between 500 and 2000 inhabitants among the surveyed population. Journeys within the same town account for 22% of the trips made. As for the rest of the destinations, the values are practically residual, with trips to another municipality included in the RMSC being the slightly higher value (6%). Therefore, trips to other municipalities other than Cáceres decrease with respect to smaller towns.

With regard to the reasons for travel, it can be seen that in all of them, there is a tacit dependence on the city of Cáceres (it is the one with the highest value in the municipalities with between 500 and 2000 inhabitants). Mainly due to the concentration and the important offer of these services in the provincial capital, figures of over 65% are obtained for journeys for study, shopping, health reasons and administrative formalities. In terms of trips for work and leisure purposes, there is a slight difference between trips within the same town and those to the city of Cáceres (although the latter is still higher). Even so, the values for journeys within the same town are around 30%.

**Figure 11:**
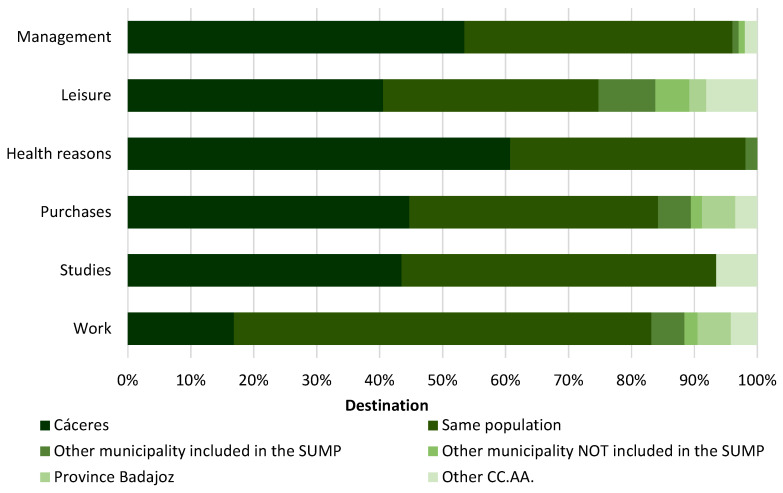
Destination of journeys of municipalities with a population of over 2000 inhabitants according to reason.

Finally, there is a third group comprising those municipalities with a population of over 2000 inhabitants (Figure 11), namely: Alcuéscar, Arroyo de la Luz, Casar de Cáceres, Garrovillas de Alconétar, Malpartida de Cáceres and Trujillo. In these municipalities, different characteristics can be observed in terms of commuting. For example, there is a strong link between the municipalities of Arroyo de la Luz, Malpartida de Cáceres and Casar de Cáceres and the city of Cáceres, which is essentially due to their proximity. On the other hand, the municipalities of Alcuéscar, Garrovillas de Alconétar and Trujillo (being further away from Cáceres) do not have such a strong link in terms of commuting compared to the rest of the municipalities in this group.

Therefore, if a comparison is made with the first and second groups, the dependence on the city of Cáceres is lower in this third group, which has a larger population (accounting for 46% of journeys). Trips within the same population suffer a considerable increase with respect to municipalities with less than 500 inhabitants and between 500 and 2000 inhabitants, accounting for 43% of trips. Similarly, journeys to other municipalities in the RMSC or other than the city of Cáceres do not exceed 5%.

If we analyse the trips by reason, we can see how the greater relevance of these municipalities within the study area and their larger size results in a considerable reduction in this dependence on the city of Cáceres. Thus, the values are similar to those for journeys within the same population. The results show a higher percentage of trips to the city of Cáceres for shopping, health, leisure and administrative purposes. However, it can be observed that trips for study purposes show similar values (around 47%). Finally, in the case of trips for work reasons, a value of 62% is reached (due to the fact that these municipalities have a greater socioeconomic dynamism). It is therefore necessary to mention that although there is still a certain dependence on the city of Cáceres in the rest of the categories, it is not as pronounced as in smaller municipalities, and it is similar to the trips made within the same municipality in several categories.

**Figure 12:**
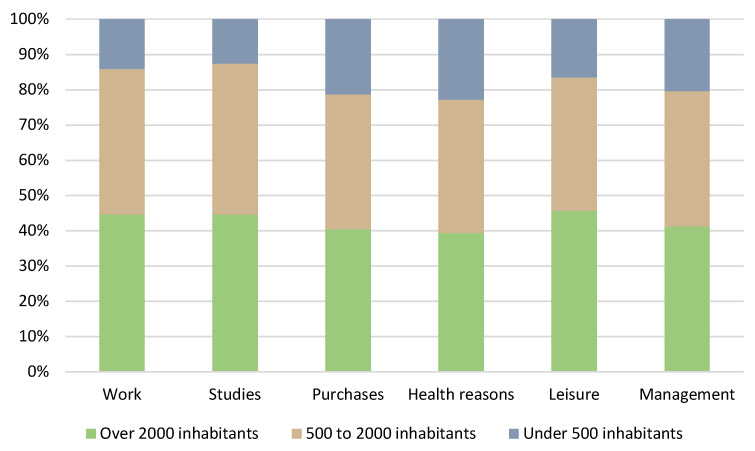
Distribution of trips by reason and population size.

In general terms, Figure 12 shows the distribution of trips in the municipalities of the study area according to population size and the reasons for the trips. Thus, it is possible to determine from which group a greater number of trips are made and what the main reasons are, since the demographic characteristics of these municipalities can determine the increase in trips in relation to the reasons for these trips.

The above figure shows how a large part of the trips are made from municipalities with a population of over 2000 inhabitants, reaching values of around 40% in the six types of reasons for travel. Among the typologies that stand out most in this group of municipalities are trips for study, work and leisure purposes, with values of around 45%. Municipalities with 500 to 2000 inhabitants show a general percentage of trips with values above 35%, in some cases exceeding 40% (work and study). With regard to the smaller municipalities, those with a population of less than 500 inhabitants, Figure 12 shows that they are the ones with the lowest number of trips in the study area, with average values of around 15%, with trips for business (20%), shopping (21%) and health (23%) standing out, due to the fact that these are the services most in demand from these population centres.

In this way, it is estimated that the destination of journeys determines the existing interrelationships between the municipalities in the study area and, therefore, is a valuable tool for finding out how mobility is established between municipalities. The fact that the city of Cáceres concentrates most of the services on offer means that the closest municipalities show a real need to make continuous journeys, which increases significantly in those municipalities with a smaller number of inhabitants and fewer services.

## 4. Discussion

The mobility diagnosis carried out in this research has made it possible to identify the degree of territorial accessibility of the city of Cáceres [75] with respect to the different municipalities that make up the RMSC as well as to the main educational and health facilities. Knowing the estimated travel time makes it possible to determine the mobility characteristics of the municipalities and thus find out which require a greater need for specific facilities and whether there is a clear relationship of dependence on the provincial capital [76]. The predominantly rural context of the municipalities in the study area means that many of them have access from lower capacity roads (regional, provincial or local), where the state of the road surface, the section of the road, or the conditions of the layout could be more limiting in comparison with high-capacity roads (national roads and motorways) [33]. The obvious difference in the typologies of the road network could lead to an increase in travel times in the case of smaller municipalities whose destination is the secondary education and health facilities of the city of Cáceres, as they are more dependent on access to these infrastructures [77]. Accessibility in rural populations has a strong impact on the mobility of their inhabitants. Thus, those rural settlements with a higher accessibility have a greater mobility and interrelation with the city of Cáceres due to their geographical location next to main communication routes [78]. However, in the same way, this is directly interrelated with the need in these population centres to travel [79] to those municipalities that have a greater supply of services, employment, health, commerce, etc.

The methodology used in the research and based on surveys of revealed preferences in order to identify the mobility of the municipalities of the RMSC has made it possible to find out the travel patterns, preferred means of transport used, main destinations and reasons for travel [5]. The specific characteristics of each municipality and its geographical location provide an attractive point of view, which directly influences the interrelations with the city of Cáceres and with the larger municipalities in the study area, as they facilitate mobility between municipalities and the attraction of services.

The results of the surveys show that the context of the municipalities of the RMSC has a significant influence on aspects related to spatial and territorial issues, which would facilitate access to certain facilities. These results are in line with what was established in [56], confirming that rural areas do not have the same range of services, which is why their inhabitants have a justified need to travel to the main centres of population for work, education, health, shopping or business. This obligation to travel implies an increase in the use of private vehicles to make these journeys, as other authors have explained [33,56]. It is clear that this means of transport is the most widely used, in addition to the fact that there is little use of public transport due to the various problems caused by it, as discussed below.

Thus, the use of associative means of transport that integrate a high number of users and are more sustainable in nature (such as public transport) did not show significant values among those surveyed, which shows that their use is very low among the population of the RMSC due to the problems associated with this means of transport, which is mainly due to the limited availability and frequency of buses operating in the study area. This model of collective mobility has not been very convincing in the study area and reflects the tacit dependence on private vehicles for any type of travel, revealing the problems that exist in the rural municipalities around the city of Cáceres [80].

It can be seen that the existing interrelationships in the study area of certain municipalities with the city of Cáceres mean that mobility plays a very important role in the quality of life of the population, since in many situations, commuting is practically obligatory (such is the case of commuters or mobile workers). The city of Cáceres is the main destination for the journeys made by the municipalities in the study area, even accounting for a larger share than the movements carried out within the same locality. These journeys are more important in the municipalities closest to the provincial capital. The greater the distance, the destination of the trips decreases in favour of other larger municipalities within the RMSC.

The importance of Cáceres as an attractor that generates continuous trips is obvious in practically all the municipalities, although the variations in the percentages of these trips depend to a large extent on the size of the population. Generally speaking, the smaller municipalities have fewer facilities and are more dependent on larger population centres (they have a concentration of schools, health centres and different public administrations). In the case of the larger municipalities, their sociodemographic and economic characteristics lead to a reduction in dependence on Cáceres. A reduction in journeys to the provincial capital can be seen, which translates into a change in the number of journeys made within the municipality itself and, to a lesser extent, those to other municipalities in the RMSC.

From the above, it is established that the use of private vehicles is predominant among the surveyed population [81], which makes it necessary to promote the use of soft means of transport within the study area, so that mobility is directed towards a sustainable model and to reduce the use of the car for shorter distances and within the municipality itself [17,82]. Although they are small municipalities (the largest does not exceed 10,000 inhabitants), the majority of journeys are made using private vehicles with a low rate of participation in soft mobility (pedestrian and cycling mobility) on the part of those surveyed. Different authors [33,57,78] state that the degree of proximity to destinations encourages mobility by active means of transport (mainly pedestrian). However, what has been observed in the study area is an excessive use of private vehicles to the detriment of other, more sustainable forms of travel. In addition, it is necessary to point out that the use of bicycles could be limited by the lack of infrastructures that provide relative safety for those inhabitants who opt for this means of transport [58].

Medium-sized urban centres and their surrounding rural areas do not have the same needs as metropolitan areas (which have more dynamic populations). On the other hand, it is worth highlighting how rural areas are absorbed by the services offered by the poles of attraction. The fact that the city of Cáceres concentrates most of the services on offer means that the closest municipalities present a real need to make continuous journeys, which increases significantly in those municipalities with fewer inhabitants and fewer services. It is necessary to emphasise that having good territorial accessibility to the health centres of the RMSC is a crucial aspect for the well-being of citizens, considering the marked ageing of the resident population in the study area. Likewise, accessibility to secondary education centres is considered a key element for the sociodemographic development of the rural municipalities in the study area, as it facilitates the revitalisation of these areas and their subsequent development. Likewise, although the needs of the municipalities are different (especially those with a markedly rural character), the ties that unite them make it advisable to jointly address shared weaknesses and take advantage of the existing resources in the study area [55]. Both the strategy promoted by the Provincial Council of Cáceres and the one established by the Regional Government of Extremadura are governance strategies that aim to lay the foundations for improving the effectiveness of public policies from a scenario favourable to coordination, through the co-responsibility of political action, as a link between institutional actors and the commitment to the whole of Extremaduran society [83] and, specifically, in this case, the 21 municipalities bordering the city of Cáceres.

In this context, the development of specific strategies for rural areas with similar problems is proposed as a future line of research in order to make it possible to achieve more sustainable journeys compatible with economic growth (enhancement of public space) and to improve the territorial connection and quality of life of the functional area. In this sense, it is considered that cross-cutting lines of work should be established such as: (a) guaranteeing people-centred mobility, practising inclusion, participation and mobility planning from a gender perspective and generational approach, (b) promoting mobility that allows equal satisfaction of the accessibility and mobility needs of rural and urban areas, as a tool to combat rural depopulation, and (c) ensuring substantial changes in people’s mobility habits to favour healthier and less polluting territories, counteracting the sources of CO_2_ from daily travel.

This research has established a diagnosis of citizen mobility patterns in a study area with eminently rural characteristics. The authors’ contribution to the advancement of knowledge has resided in the combination of three methodological aspects: (a) obtaining and analysing data in open format from public repositories, (b) applying GIS tools, and (c) carrying out revealed preference surveys, which were carried out in an inter-municipal area of a dispersed territorial nature and whose population is clearly ageing. It is considered that the results obtained could serve as a starting point for other research carried out in similar regions.

## 5. Conclusions

This article has addressed the diagnosis of existing mobility in the RMSC through the use of revealed preference surveys and territorial accessibility analysis (two independent approaches). Firstly, geographic information system tools (network analysis) have been used to determine the access time from the municipalities in the study area to the main basic services and, secondly, the mobility patterns of the inhabitants have been examined by analysing the population’s responses in the surveys carried out. In this way, this research is the first step towards identifying trends in the movement of the population under study, making it possible to determine the existing problems and, through future work, to explore different solutions that are in line with the objectives established by the different public administrations.

The main conclusion of the research is the existence of an unbalanced modal split, with a preponderance of private vehicle use (regardless of the destination or the reason for the journey). In the municipalities as a whole, the average rate of private vehicle use is over 75%. With reference to other means of transport, the bus is used in only 6% of cases, with a surprisingly low percentage of journeys made on foot (only 15%). On the other hand, the city of Cáceres appears in the preference surveys revealed as the main destination of the journeys made (a real attractor or generator of journeys), even more so than internal journeys within each of the municipalities. In addition, there is a very weak inter-municipal connection dynamic (only 6% of the most frequent trips were to another municipality in the RMSC).

## Figures and Tables

**Table 1:** Population groups of municipalities.

Size	Municipalities	Population
Under 500 inhabitants	Botija, Casas de Don Antonio, Herreruela, Plasenzuela, Santa Marta de Magasca and Santiago del Campo	1694
500 to 2000 inhabitants	Aldea del Cano, Aliseda, Brozas, Montánchez, Sierra de Fuentes, Talaván, Torremocha, Torreorgaz and Torrequemada	11,664
Over 2000 inhabitants	Alcuéscar, Arroyo de la Luz, Casar de Cáceres, Garrovillas de Alconétar, Malpartida de Cáceres and Trujillo.	28,022

**Table 2:** Survey sample by category.

Category	Subcategory	N	%
Gender	Male	105	35.71%
	Woman	187	63.61%
	I prefer not to say	2	0.68%
Age	20–29	26	8.84%
	30–39	43	14.63%
	40–49	75	25.51%
	50–59	85	28.91%
	60–69	37	12.59%
	70–79	24	8.16%
	80–89	4	1.36%
Employment status	In retirement or early retirement	52	17.75%
	Unemployed	29	9.90%
	Studying	8	2.73%
	Other situation or inactivity	13	4.44%
	Household chores	18	6.14%
	Working	174	59.04%

## Data Availability

https://pmuscaceres.es/ (accessed on 5 January 2023).
